# Reactive Oxygen Species Deficiency Due to Ncf1-Mutation Leads to Development of Adenocarcinoma and Metabolomic and Lipidomic Remodeling in a New Mouse Model of Dextran Sulfate Sodium-Induced Colitis

**DOI:** 10.3389/fimmu.2018.00701

**Published:** 2018-05-14

**Authors:** Lina Carvalho, Joana R. M. Gomes, Ludgero C. Tavares, Ana R. Xavier, Karel D. Klika, Rikard Holmdahl, Rui A. Carvalho, M. Margarida Souto-Carneiro

**Affiliations:** ^1^Faculty of Medicine, Institute of Anatomic Pathology, University of Coimbra, Coimbra, Portugal; ^2^Center for Neuroscience and Cell Biology, University of Coimbra, Coimbra, Portugal; ^3^Molecular Structure Analysis Department, Deutsches Krebsforschungszentrum (DKFZ), Heidelberg, Germany; ^4^Department of Medical Biochemistry and Biophysics, Karolinska Instituite (KI), Stockholm, Sweden; ^5^Department of Life Sciences, Faculty of Science and Technology, Center for Functional Ecology, University of Coimbra, Coimbra, Portugal; ^6^Department of Rhematology, Medical Clinic 5, Universitätsklinikum Heidelberg, Heidelberg, Germany

**Keywords:** reactive oxygen species, dextran sulfate sodium, adenocarcinoma, colitis, metabolism, nicotinamide adenine dinucleotide phosphate oxidase, nuclear magnetic resonance, lipids

## Abstract

Inflammatory bowel disease is characterized by chronic relapsing idiopathic inflammation of the gastrointestinal tract and persistent inflammation. Studies focusing on the immune-regulatory function of reactive oxygen species (ROS) are still largely missing. In this study, we analyzed an ROS-deficient mouse model leading to colon adenocarcinoma. Colitis was induced with dextran sulfate sodium (DSS) supplied *via* the drinking water in wild-type (WT) and Ncf1-mutant (Ncf1) B10.Q mice using two different protocols, one mimicking recovery after acute colitis and another simulating chronic colitis. Disease progression was monitored by evaluation of clinical parameters, histopathological analysis, and the blood serum metabolome using ^1^H nuclear magnetic resonance spectroscopy. At each experimental time point, colons and spleens from some mice were removed for histopathological analysis and internal clinical parameters. Clinical scores for weight variation, stool consistency, colorectal bleeding, colon length, and spleen weight were significantly worse for Ncf1 than for WT mice. Ncf1 mice with only a 7-day exposure to DSS followed by a 14-day resting period developed colonic distal high-grade dysplasia in contrast to the low-grade dysplasia found in the colon of WT mice. After a 21-day resting period, there was still β-catenin-rich inflammatory infiltration in the Ncf1 mice together with high-grade dysplasia and invasive well-differentiated adenocarcinoma, while in the WT mice, high-grade dysplasia was prominent without malignant invasion and only low inflammation. Although exposure to DSS generated less severe histopathological changes in the WT group, the blood serum metabolome revealed an increased fatty acid content with moderate-to-strong correlations to inflammation score, weight variation, colon length, and spleen weight. Ncf1 mice also displayed a similar pattern but with lower coefficients and showed consistently lower glucose and/or higher lactate levels which correlated with inflammation score, weight variation, and spleen weight. In our novel, DSS-induced colitis animal model, the lack of an oxidative burst ROS was sufficient to develop adenocarcinoma, and display altered blood plasma metabolic and lipid profiles. Thus, oxidative burst seems to be necessary to prevent evolution toward cancer and may confer a protective role in a ROS-mediated self-control mechanism.

## Introduction

Inflammatory bowel disease (IBD) is characterized by chronic relapsing idiopathic inflammation of the gastrointestinal tract. The two major known forms of IBD are Crohn’s disease and ulcerative colitis (UC). Discontinuous transmural lesions may appear in any segment of the gastrointestinal tract in Crohn’s disease whereas UC is restricted to lesions in the colon and rectum mucosae. These two separate conditions have distinct clinical, endoscopic, and histological profiles though they share some overlapping clinical features (1–7). In both cases, there are no definitive treatments and the active and remission cycles are managed with anti-inflammatory or immunomodulatory drugs and eventually surgery, compromising the patients quality of life (8). Perhaps one of the most pressing factors for routine monitoring is the heightened risk of cancer development in chronic inflammatory lesions, namely colorectal carcinoma and small bowel adenocarcinoma (9–11). Regarding colorectal carcinoma, two major forms are pathophysiologically distinguishable, sporadic colorectal cancer, and colitis-associated colorectal cancer. Although they share common pathogenic elements, their progression follows different molecular mechanisms (12, 13). Tumor-promoting inflammation and genomic predisposition and instability provide favorable ground for cancer onset while the deregulation of cellular bioenergetics and immune-evasion have recently been identified as emerging hallmarks (14). Even though the complexity of the thematic does not allow the pinpoint of a single root cause of the problem, evidence from research suggests a pivotal role for oxygen and reactive oxygen species (ROS) in carcinogenesis, particularly in promoting inflammation, causing oxidative DNA damage, altering signaling pathways and modulating metabolism and immune response (14, 15). Regular cellular function will generate ROS but the balance between pro- and antioxidants is a delicate one. This homeostatic regulation may be achieved through several scavenging processes, namely superoxide dismutase, glutathione and catalase, among others. Excessive oxidative stress may cause extensive damage and signal cell death either by necrosis or apoptosis, but on the other hand, homeostatic levels regulate many signal transduction pathways and promote cell proliferation and survival (16, 17). Additionally, ROS generation is of seminal importance for immune responses to pathogens, in particular, bacteria and fungi. In granulocytes and macrophages, phagocytic activity generates an oxidative burst from superoxide anion production by NADPH oxidase complex 2 (NOX2), which is then dismutated into peroxide and other reactive species toxic to bacteria (18). ROS function equally as immunological regulators, preventing chronic inflammation and autoimmunity (19).

Reactive oxygen species imbalances also contribute indirectly to the aerobic glycolysis that generally characterizes cancerous cells—the Warburg effect. Hypoxia and increased ROS levels inhibit prolyl hydroxylases, which lead to hypoxia-inducible transcription factor (HIF-1α) stabilization and subsequent gene expression thus triggering upregulation of glucose transporters and glycolytic enzymes essential to aerobic glycolysis (20). Other signaling pathways may play a role in modulating metabolism in cancer cells, such as activation of the c-Myc transcription factor or the oncogene *KRAS*, or the loss of function of the tumor suppressor gene *P53* (17). Cancerous cells may also exhibit adaptation to the heightened oxidative stress by upregulating ROS-scavenging enzymes, controlling excessive protein/DNA damage, and lipid peroxidation, thereby avoiding cell death (17).

A set of contributing factors are required to develop IBD, namely genetic predisposition, detrimental gut microbiota, defective mucosal barrier function, exacerbated immune response, and environmental triggers (6). Unresolved inflammation may develop dysplasia and eventually favor cancer onset (21). Interestingly, a rare inheritable disease, chronic granulomatous disease (CGD) characterized by defective NOX2, renders phagocytes unable to produce superoxide anion (22). In fact, CGD patients frequently have an associated IBD and exhibit Crohn-like symptoms (22, 23). Research on the link between IBD and cancer has been using several genetically engineered mice models prone to colorectal cancer whereby colitis is chemically induced by dextran sodium sulfate (DSS) supplied *via* the drinking water (24).

Studies focused on the immune-regulatory function of ROS and how their deficiency impacts metabolism and inflammation-mediated tumorigenesis, surprisingly, are still largely missing. B10.Q mice with a point mutation in the p47 NOX subunit [Ncf1-mutant (Ncf1)/p47^phox^] lack ROS production leading to deficient T-cell tolerance induction, triggering autoimmunity with a type I interferon signature [reviewed in Ref. (19)]. The absence of ROS in these mice led to poor recovery from two cycles of acute DSS-induced colitis and was characterized by extensive nitric oxide-dependent mucosal inflammation and dysplasia (25). The oral administration of DSS to rodents induced colonic inflammation that was clinically and histologically similar to human UC (25). Like the human disease, DSS-induced chronic UC is complicated by the development of colorectal dysplasia and adenocarcinoma (26, 27). Therefore, in the present study, B10.Q/Ncf1 mice were used to develop a new model of inflammation-driven colon carcinoma by induction of colitis with DSS and address the putative role of ROS in tumor formation and systemic metabolic alterations.

## Materials and Methods

### Animals

Male and female homozygous Ncf1 (BQ.*Ncf1^m1J/m1J^*, abbreviated to Ncf1, *n* = 30) and wild-type (WT, *n* = 30) B10.Q mice between 6 and 8 weeks old were obtained from breeding heterozygous mice followed by genotyping as previously described (28). Animals were bred and maintained under standard conditions at the specific pathogen-free animal facility of the Faculty of Pharmacy, University of Coimbra with food and water supplied *ad libitum* within a controlled temperature environment, and alternating 12-h light/dark cycles. All animal studies were approved by the internal FFUC Animal Facility Ethics Committee and were in accordance with EU legislation for experimental animal welfare.

### Induction of Colitis

Colitis was induced by oral administration of 3% w/v DSS (average mol. wt. 40,000 g/mol, AppliChem, Darmstadt, Germany) in the first induction period and 2.5% w/v DSS in the second induction period *via* drinking water supplied *ad libitum*. The DSS concentration was reduced for the second cycle to prevent premature death so that animals survived till the end of the experiment. DSS reduction also minimized weight loss and rectal bleeding, thus limiting excessive suffering.

Two different colitis-induction protocols were used: in protocol remission (r), mice were subjected to a 7-day DSS induction followed by 21 days of resting on normal water; in protocol induction (i), mice were subjected to 7 days of DSS induction, followed by 14 days of resting on normal water and then a second 7-day DSS-induction period.

### Clinical Evaluation

Animals were monitored every third day for alterations in colitis-related clinical scores: weight variation, stool consistency, and colorectal bleeding. Pain was monitored on a daily basis through observation of animal activity for 5 min during the resting (light cycle) and active (dark cycle) periods. Blood and stool scorings were performed as previously described (25). In brief, blood scoring: 0- no blood; 1- visible blood; 2- rectal bleeding; stool consistency scoring: 0- normal; 1- soft but formed; 2- very soft; 3- diarrhea. Five mice from each group were sacrificed by cervical dislocation under anesthesia at three experimental time points: days 0 (baseline controls), 22 and 30. Tissues were collected for histopathological analysis (*vide infra*) and assessment of colon length and spleen weight.

### Histopathological Evaluation of Colitis

Swiss rolls of the whole colon, rolled from the rectum to cecum, were formalin fixed and paraffin embedded. Sections were hematoxylin/eosin (HE) stained according to standard protocols.

Immunohistochemistry was performed on distal colon sections. In brief, endogenous peroxidase activity was quenched by 15 min incubation with 3% diluted hydrogen peroxide. Non-specific binding was blocked with Ultra V Block (Ultra Vision Kit; TP-125-UB; Lab Vision Corporation, Fremont, CA, USA). Incubation with the primary rabbit monoclonal antibody against β-catenin (clone D10A8, Cell Signaling Technology Europe, Frankfurt am Main, Germany) was followed by incubation with biotin-labeled secondary antibody (Ultra Vision Kit; TP-125-BN; Lab Vision Corporation, Fremont, CA, USA). Primary antibody binding was localized in tissues using peroxidase-conjugated streptavidin (Ultra Vision Kit; TP-125-HR; Lab Vision Corporation, Fremont, CA, USA) and 3,3-diaminobenzidine tetrahydrochloride (RE7190-K; Novocastra Laboratories Ltd., Newcastle, UK) was used as chromogen, according to manufacturer’s instructions. Hematoxylin was used to counterstain the slides.

Inflammation was scored in proximal and distal segments halves regarding the number of inflammatory foci: 0- no inflammatory focus; 1- one inflammatory focus; 2- two inflammatory foci; 3- three or more inflammatory foci. Lymphocytes, plasma cells, and neutrophils infiltration were scored based on cell morphology, with approximate percentage validation of each subset within the total observed inflammatory cells in inflammatory foci, as previously described (25).

Epithelial morphology was scored based on the presence of tubular and villous patterns: tubular-glandular hyperplasia above the *muscularis mucosae* and villous formation of superficial villous projections. Dysplasia was scored in the same segments using a semi-quantitative scale according to the World Health Organization (WHO) 2010 guidelines for colon adenocarcinoma classification (26): 0- no dysplasia; 1- hyperchromatic nuclear pluristratification and *lamina propria* separated glands; 2- epithelial low-grade dysplasia (complex ramified glands with cell hyperplasia and pluristratified hyperchromatic nuclei); 3- epithelial high-grade dysplasia (beyond low-grade dysplasia, nuclear atypia, mitosis and reduced *lamina propria* area).

### Nuclear Magnetic Resonance (NMR) Spectroscopic Analysis

Blood serum from mice was collected for metabolic profiling using^1^ H NMR spectroscopy. Samples consisted of 70 µL of sera plus 70 µL of D_2_O (99.9%) together with 35 µL of sodium fumarate (10 mM) and phosphate buffer dissolved in D_2_O (99.9%) for use as an internal reference. Samples (total volume 175 µL) were placed into 2.5 mm NMR tubes and spectra acquired using a 600 MHz Bruker NMR spectrometer equipped with an inverse configuration probe. For all samples, regular ^1^H acquisition with presaturation (sw = 10 kHz, ns = 32, td = 60 k, aq = 3 s) and Carr–Purcell–Meiboom–Gill (CPMG) (28) (sw = 10 kHz, ns = 350, spin-echo time = 200 168 ms, td = 60 k, aq = 3 s) spectra were acquired. Spectra were processed with TopSpin using 0.2 Hz of line broadening and manual phasing while AMIX was used for metabolite assignment and multivariate statistics. For metabolomics integration, spectra were subjected to bucketing by AMIX from 0.5–9 ppm (excluding the solvent region) with the spectral area normalized to the sum of all points. All metabolomics data have been deposited to the EMBL-EBI MetaboLights database (DOI: 10.1093/nar/gks1004. PubMed PMID: 23109552) with the identifier MTBLS593.

### Statistical Analysis

All data were tested for normal distribution with Levene’s test. Since data did not follow a normal distribution, the non-parametric Kruskal–Wallis test followed by a non-parametric Mann–Whitney *U* test were used to compare values between groups and time points using GraphPrism 6.01 (GraphPad Software Inc., CA, USA).

Statistical differences between curves were determined using a two-sided hypothesis permutation test with 10,000 permutations (http://bioinf.wehi.edu.au/software/compareCurves/index.html) (29). Bivariate correlation studies (Spearman, two-sided) were performed using SPSS 17. Differences were considered significant for *p* < 0.05. For NMR metabolomics analysis, data were analyzed using MetaboAnalyst software performing interquartile range filtering, log transformation, and pareto scaling prior to principal component analysis (PCA) or partial least squares–discriminant analysis (PLS–DA) modeling (30).

## Results

### Clinical Signs of DSS-Induced Colitis

**Figure 1:**
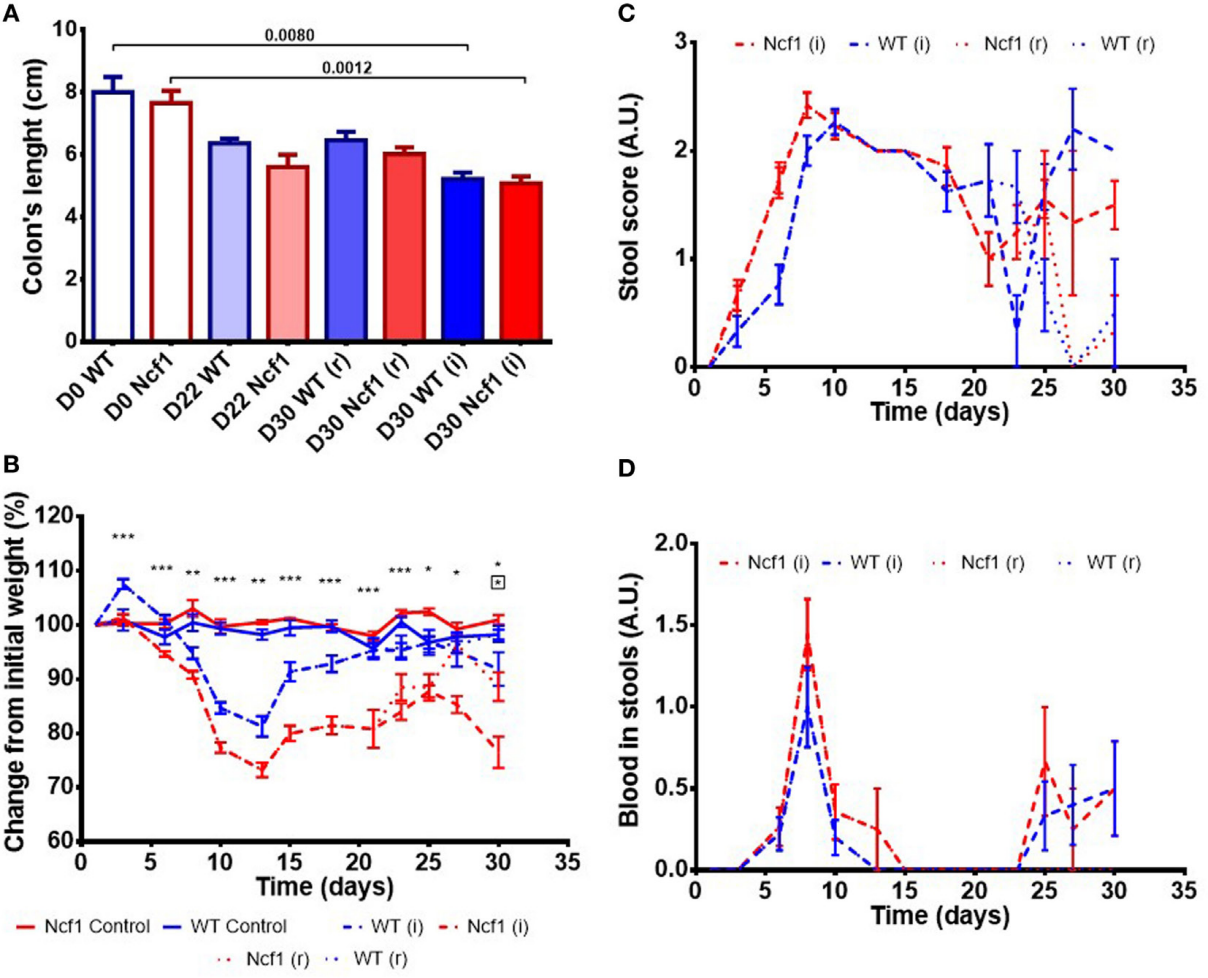
Ncf1-mutant (Ncf1) mice presented more severe clinical scores than their wild-type (WT) counterparts. **(A)** Colon lengths in centimeters for days 0, 22, and 30 of control, recovery (r), and twice colitis-induced (i) WT and Ncf1 groups. **(B)** Change in body weights from the initial average baseline weight over the experimental period, average weight ± SE (baseline weights: WT = 29.8 ± 3.90 g, Ncf1 = 31.3 ± 3.9 g). Differences between groups and time points were calculated by two-way ANOVA. **(C)** Mice stool consistency score after colitis induction with dextran sulfate sodium (DSS). **(D)** Colorectal bleeding score after colitis induction. Weight, stool consistency, and colorectal bleeding scorings are detailed in Section “Materials and Methods.”. Asterisks indicate significant differences: *p < 0.05, **p < 0.001, ***p < 0.0001 between WT and Ncf1 mice receiving DSS; boxed asterisks indicate significant differences: *p < 0.05 between Ncf1 recovery (r) and induction (i) groups.

Colitis induction with DSS led to a reduction in the colon length in both the WT and the Ncf1 mice resulting in a significant shortening of the colon after a second induction period (Figure 1A).

Body weight was monitored throughout the study (Figure 1B). At baseline, the weights of Ncf1 and WT mice were comparable (WT = 29.8 ± 3.90 g, Ncf1 = 31.3 ± 3.9 g). Weight loss began on day 3 after DSS colitis induction with all DSS-treated animals reaching the minimum weight on day 13 (i.e., during the recovery period) and with Ncf1 mice presenting a greater weight loss than WT mice. In protocol (r), the WT mice recovered their baseline weight while Ncf1 only recovered, at most, up to 90% of their original weight. The mice which were subjected to a second cycle of DSS-induced colitis [protocol (i)], partially recovered their weight until day 25 when a new phase of weight loss set in. Ncf1 mice suffered greater weight loss (reduction to 75% of baseline weight) than the WT mice (reduction to only 90% of baseline weight). While WT mice on both protocols had comparable weights until the end of the experimental period, Ncf1 mice under the (i) protocol had a greater weight loss than those under the (r) protocol.

The presence of colorectal blood and the consistency of the feces are two further clinical signs of DSS-induced colitis. During the first induction period, both Ncf1 and WT groups had decreased stool consistency and increased anal bleeding. During the resting period, both groups started to recover with respect to these two clinical parameters. WT and Ncf1 mice submitted to protocol (i) both presented a new surge in anal bleeding together with softer stools, these clinical symptoms were significantly different to those animals undergoing protocol (r) (Figures 1C,D).

### Histopathologic Assessment of DSS-Induced Colitis

To evaluate epithelial morphology, inflammation, and dysplasia, the defined colon histopathology scores were applied using untreated WT and Ncf1 animals as controls and registered after colitis induction at day 22 for both experiments and day 30 for protocol r and protocol i.

**Figure 2:**
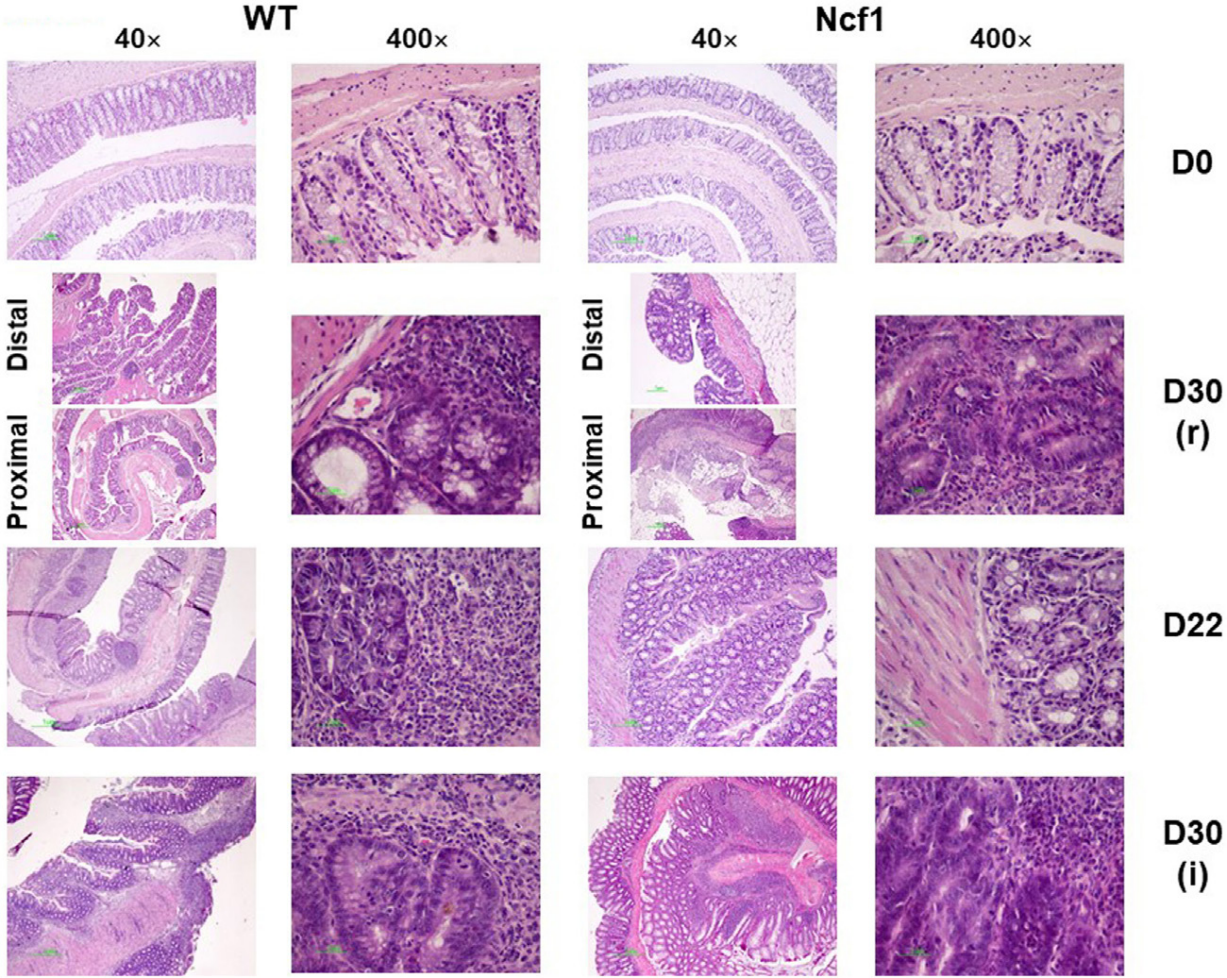
Colonic mucosa of control, recovery (r), and twice colitis-induced (i) wild-type (WT) and Ncf1-mutant (Ncf1) groups. *Day 0*: glands are smaller, fewer, and with small epithelial cell nuclei in Ncf1 mice colon compared with WT mice. *Day 30 of recovery*: WT mouse colon with basal small gland hyperplasia and villous epithelial adaptation; Ncf1 mouse colon with superficial villous adaptation, high-grade dysplasia, and intramucosal adenocarcinoma. *Day 22 for both experiments*: both WT and Ncf1 mice colons with superficial villous glandular hyperplasia, and inflammation-reactive atypia in WT mouse colon; low-grade dysplasia; and mucinous cells hyperplasia in tubular glands of WT mouse colon contrasting to basal high-grade dysplasia in Ncf1 mouse colon. *Day 30 of the second colitis induction*: Superficial villous mucosae and basal cell glandular persistence with high-grade dysplasia in WT mouse colon compared with the superficial villous atrophy and well-differentiated adenocarcinoma in Ncf1 mouse colon. hematoxylin/eosin staining with 40× and 400× magnifications.

At baseline, WT mice had well defined glands above *muscularis mucosae*, whereas Ncf1 mice presented a reduced number of glandular tubules (Figure 2 D0), both with the absence of dysplasia and inflammation.

**Figure 3:**
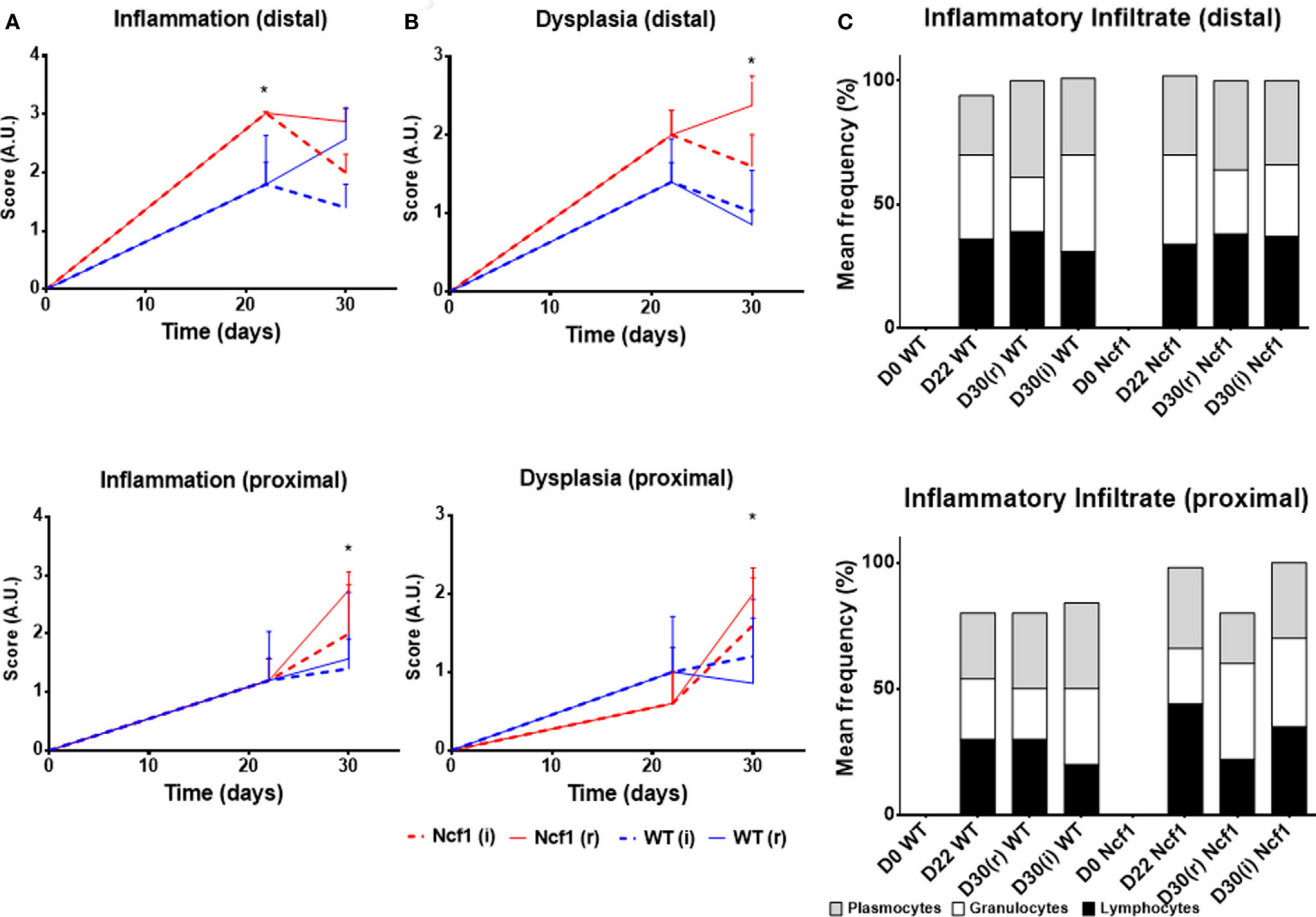
Histological evaluation of inflammation **(A)**, dysplasia **(B)**, and cellular composition of the inflammatory infiltrate **(C)** at distal and proximal segments of the colon for Ncf1-mutant (Ncf1)* mice, and WT mice. Asterisks indicate *p* < 0.05, Mann–Whitney *U* test between Ncf1(r) and WT (r) and Ncf1(i) and WT(i). Inflammation and dysplasia scoring systems are detailed in Section “Materials and Methods.”

On day 22, WT mice maintained preserved epithelial morphology and superficial villous projections with low-grade distal dysplasia (1.4 ± 0.2) and low-distal inflammation (1.8 ± 0.4). The Ncf1 group also presented villous projections, but these were formed by compacted glands with less interstitial vascularization in addition to glandular higher grade dysplasia (2.0 ± 0.3, *p* = 0.5) and foci of well-differentiated adenocarcinoma accompanied by higher distal inflammation (3.0 ± 0.0, *p* = 0.039) (Figure 2 D22; Figure 3). Even though Ncf1 inflammatory score was higher than the WT, the cellular composition of the inflammatory infiltrates had similar frequencies of grnaulocytes, lymphocytes, and plasmacytes in both groups. Proximal inflammation and dysplasia were low in both groups.

**Figure 4:**
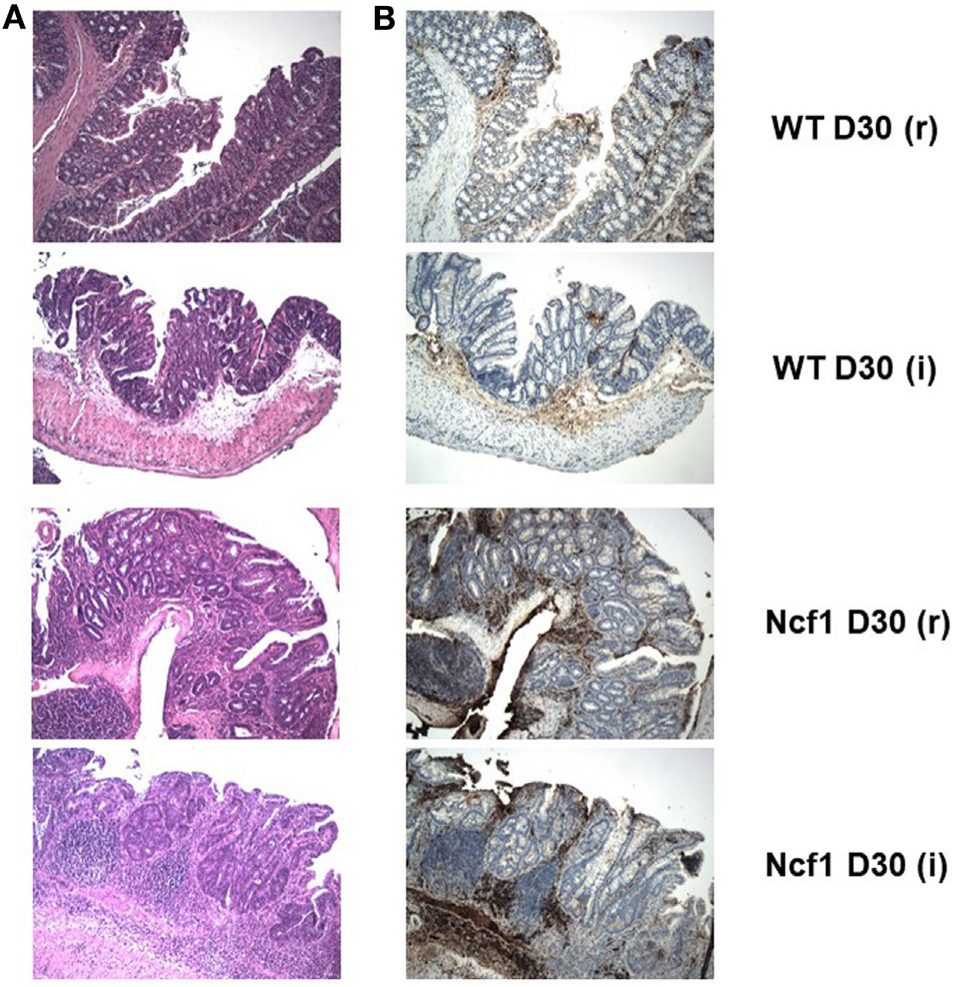
The extensive inflammatory infiltrates present in the recovery (r) and twice colitis-induced (i) Ncf1-mutant (Ncf1) mouse colons harbor lymphocytes expressing high levels of β-catenin. In wild-type (WT) mice, some inflammatory foci contain scattered β-catenin-expressing lymphocytes. **(A)** Hematoxylin/eosin staining of representative colon sections of each protocol at D30 with 100× magnification; and the corresponding **(B)** β-catenin staining (brown) with 100× magnification. The colon sections are from the same samples as Figure 2, thus corresponding to the description of the histological features in that legend.

On day 30 for protocol r [Figure 2 D30(r); Figures 3 and 4 D30(r)], WT mice maintained villous projections corresponding to half of the *mucosae* length, supported by tubular-glandular hyperplasia above the *muscularis mucosae*, with different sizes and segments of very small glands with low-grade dysplasia foci. Scattered lymphocytes in small inflammatory infiltrates expressed β-catenin. In Ncf1 mice colons, there were a reduced number of glands under villous projections in the colon, although the morphology was similar to day 22. In general, Ncf1 mice had more high-grade dysplasia (with anisocariosis with persistent nucleoli and visible mitosis) and foci of well-differentiated adenocarcinoma with large inflammatory infiltrates rich in β-catenin-expressing lymphocytes (total mean scores for dysplasia WT = 2.20 ± 0.49, Ncf1 = 3.20 ± 0.74, *p* = 0.42; total mean scores for inflammation WT = 2.80 ± 0.66, Ncf1 = 4.00 ± 0.95, *p* = 0.31).

On day 30 for protocol i [Figure 2, Day 30(i); Figures 3 and 4 D30(i)], a persistent adaptation of the colonic sections was observed. WT colon presented superficial epithelial villous projections without extensive inflammation. In distal segments, high-grade dysplasia persisted in both groups. Ncf1 proximal and distal colonic segments maintained a glandular morphology with hyperchromatic nuclei and mucosa-associated lymphoid tissue hyperplasia. Additionally, Ncf1 colon developed invasive well-differentiated adenocarcinoma in segments where narrower, reserve microglands were visible above the *mucularis mucosae*, invading till the *muscularis propria*, accompanied by extensive inflammatory infiltrates harboring lymphocytes expressing high levels of β-catenin (mean scores for total dysplasia WT = 1.71 ± 0.52, Ncf1 = 4.38 ± 0.26, *p* = 0.001; mean scores for total inflammation WT = 4.14 ± 0.51, Ncf1 = 5.25 ± 0.31, *p* = 0.03).

Considering the dysplasia score in WT vs Ncf1 mice, we found a very strong positive correlation between dysplasia score and inflammation score (*r_s_* = 0.835) in Ncf1 mice, while this correlation was only moderate for the WT mice (*r_s_* = 0.591) (Table 1). Moderate-to-strong positive correlations could be observed for the infiltrating leukocytes in both groups (Table 1). The dysplasia score is also negatively correlated with the colon length in both groups, consistent with the previous results in Figure 1A.

**Table 1:** Spearman correlations and *p*-values for dysplasia score vs inflammation score, frequency of leukocytes infiltrating the colon mucosa and colon length.

Spearman correlations		Dysplasia score		
		All groups (*n* = 45)	Ncf1 (*n* = 22)	WT (*n* = 23)
Inflammation score	Correlation coefficient	0.838**	0.835**	0.591**
	Sig. (two-tailed)	0.000	0.000	0.003
Lymphocytes (%)	Correlation coefficient	0.527**	0.489*	0.556**
	Sig. (two-tailed)	0.000	0.021	0.006
Granulocytes (%)	Correlation coefficient	0.676**	0.745**	0.595**
	Sig. (two-tailed)	0.000	0.000	0.003
Plasmocytes (%)	Correlation coefficient	0.446**	0.460*	0.473*
	Sig. (two-tailed)	0.002	0.031	0.023
Colon length	Correlation coefficient	−0.574**	−0.507*	−0.452*
	Sig. (two-tailed)	0.000	0.016	0.035

The table summarizes the significant correlations considering all groups together and Ncf1-mutant (Ncf1) vs wild-type (WT) groups. Significant correlations are indicated by asterisks: *p < 0.05; **p < 0.01.

### Metabolic Profile of Blood Serum of DSS-Induced Colitis Mice

**Figure 5:**
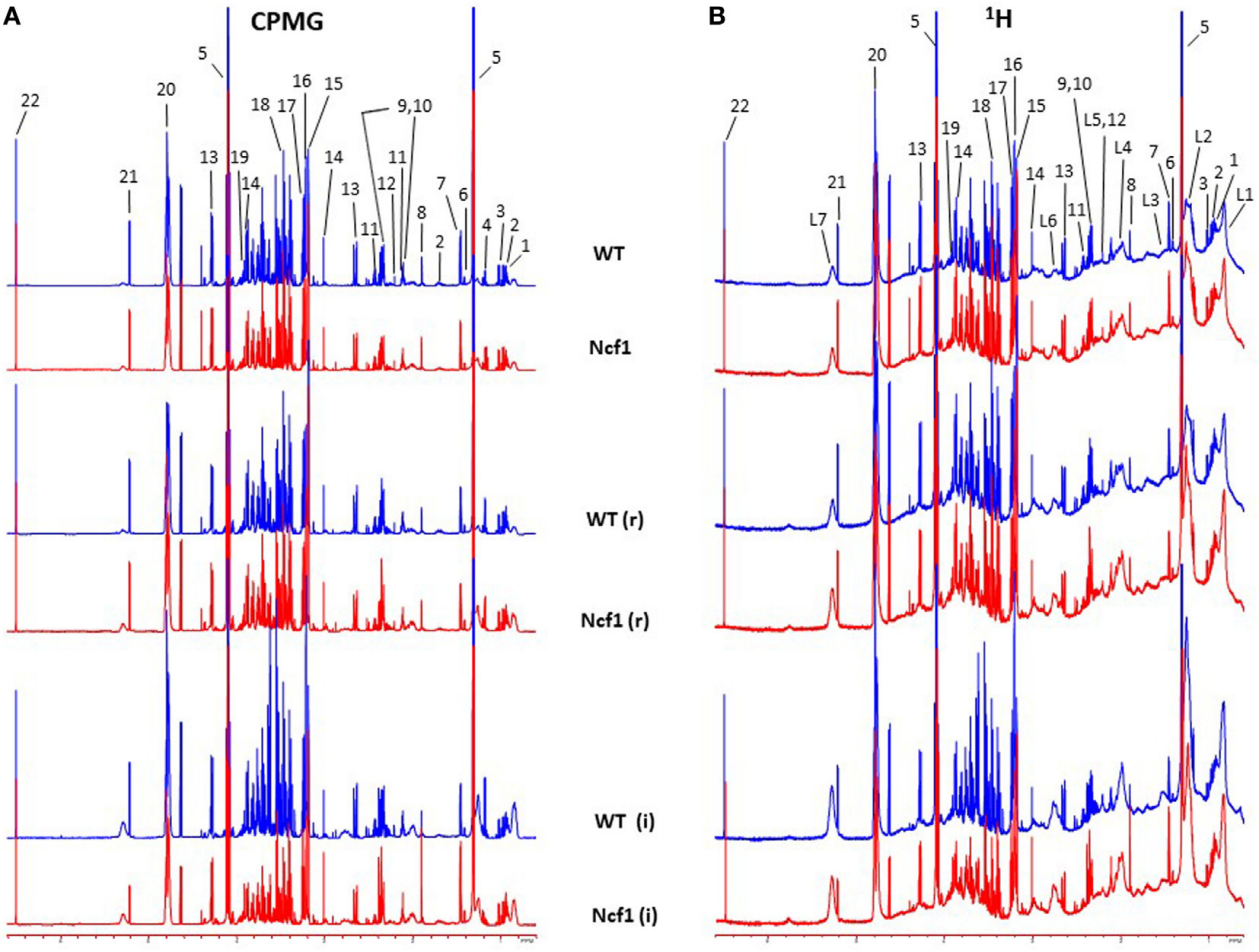
Representative Carr–Purcell–Meiboom–Gill (CPMG) **(A)** and ^1^H **(B)** nuclear magnetic resonance spectra of blood sera from wild-type (WT) and Ncf1-mutant (Ncf1) mice at baseline, after recovery of the first period of dextran sulfate sodium (DSS)-induced colitis (r) and after the second period of DSS-induced colitis (i). The CPMG pulse sequence suppresses the broad signals from macromolecules allowing a better characterization of the signals arising from the small metabolites. Signal assignments: (1) isoleucine, (2) leucine, (3) valine, (4) β-hydroxybutyrate, (5) lactate, (6) threonine, (7) alanine, (8) acetate, (9) proline, (10) glutamate, (11) glutamine, (12) methionine, (13) malate, (14) creatine, (15) choline, (16) phosphorylcholine/glycerophosphocholine, (17) taurine, (18) glycine, (19) serine, (20) water, (21) α-glucose, (22) fumarate, (L1) lipid methyls, (L2) lipid aliphatic chain, (L3) lipid β-methylenes, (L4) lipid allylic methylenes, (L5) lipid α-methylenes, (L6) lipid polyunsaturated allylic methylenes, and (L7) lipid alkenes.

**Figure 6:**
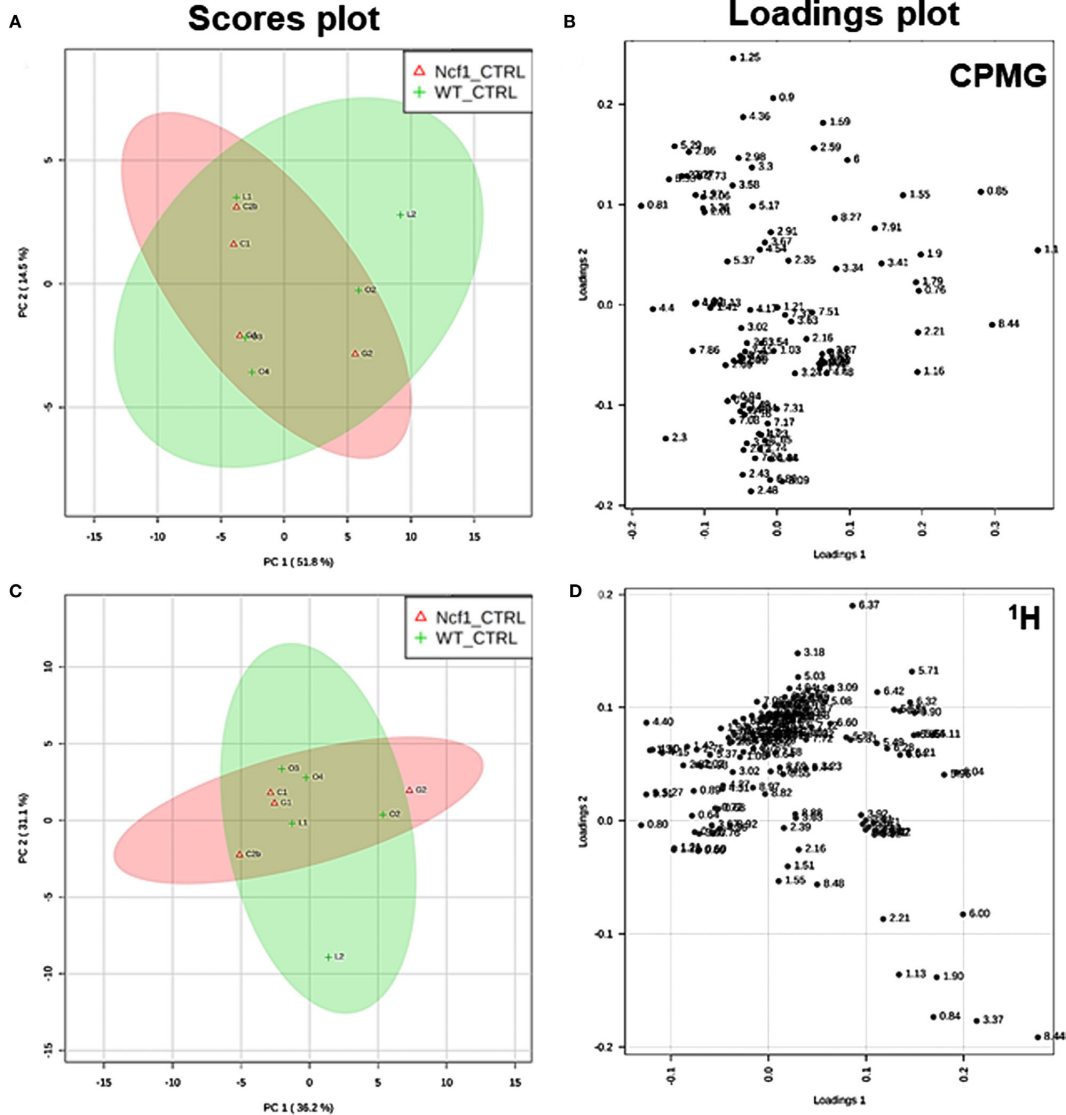
Control wild-type (WT) and Ncf1-mutant (Ncf1) mice display similar metabolomic profiles showing no difference in small molecule metabolites and lipid and macromolecular fractions. The scores plot **(A)** accounts for 66.3% of the total variance (PC1 = 51.8%, PC2 = 14.5%) and the loadings plot **(B)** shows the δ of each variable (bucket) for the principal component analysis (PCA) analysis of Carr–Purcell–Meiboom–Gill (CPMG) spectra of Ncf1 and WT control groups. The CPMG pulse sequence suppresses the broad signals from macromolecules allowing analyze small molecule metabolites. The scores plot **(C)** summarizes 67.3% of the total variance (PC1 = 36.2%, PC2 = 31.1%) and the loadings plot **(D)** shows the δ of each variable (bucket) of the PCA analysis of ^1^H spectra of Ncf1 vs WT control groups. The ^1^H spectra allow analysis of the aqueous lipid fraction and other macromolecules.

**Figure 7:**
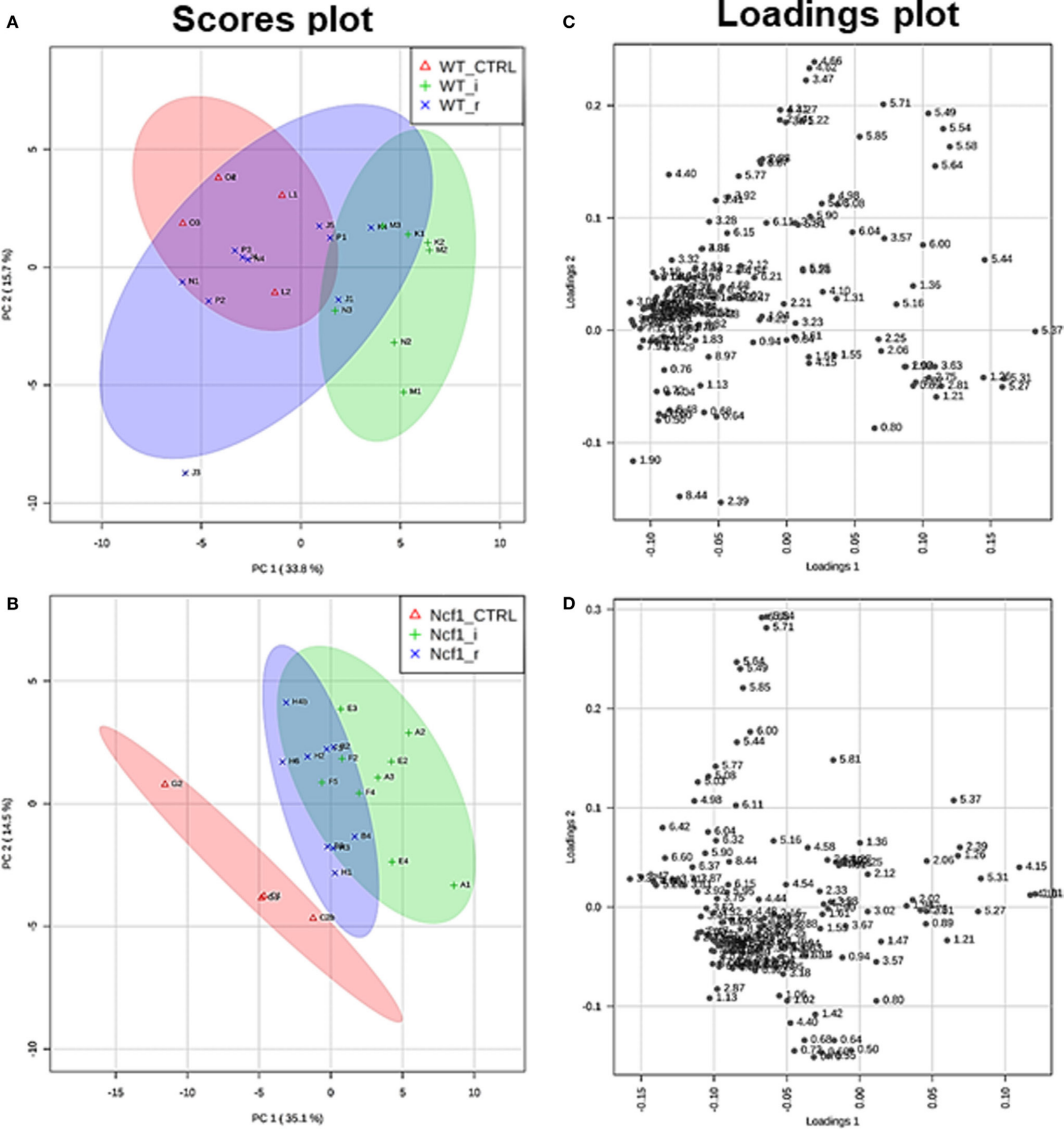
The presence of the Ncf1-mutant (Ncf1) mutation leads to distinct metabolomic profiles in mice either recovering from (r) or after a second period of DSS-induced colitis (i). This contrasts with wild-type (WT) mice which present overlapping metabolomic profiles between control, recovery, and second colitis-induced groups. Principal component analysis of ^1^H spectra of WT and Ncf1mice. The scores plot for the WT reaches 49.4% of the total variance (PC1 = 33.8%, PC2 = 15.6%) **(A)**; for the Ncf1 achieves 49.6% of the total variance (PC1 = 35.1%, PC2 = 14.5%) **(B)**. The loadings plot shows the δ of each variable (bucket) for the WT **(C)** and the Ncf1 **(D)** groups.

**Figure 8:**
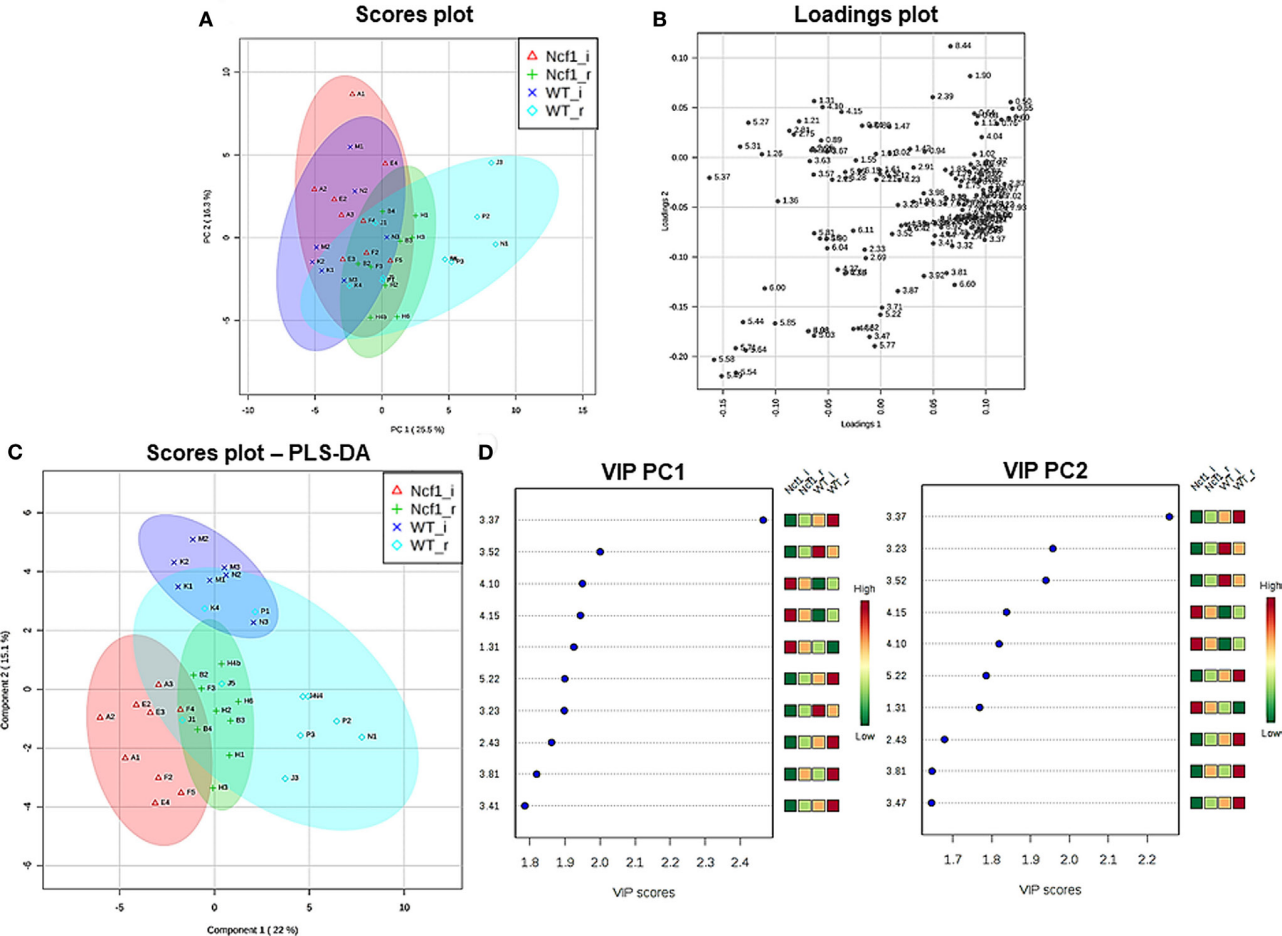
The systemic metabolic profile after a second period of dextran sulfate sodium-induced colitis is markedly distinct between mice bearing the Ncf1-mutation (Ncf1) and the wild-type (WT) group. However, the two recovery groups tend to diverge from the induced groupings and edge toward a common metabolic profile. Principal component analysis (PCA) and partial least squares–discriminant analysis (PLS–DA) analysis of ^1^H spectra of Ncf1 vs WT after the second colitis induction (i) and recovery (r) groups. The PCA scores plot explains 41.8% of the total variance (PC1 = 25.5%, PC2 = 16.3%) **(A)** and the loadings plot shows the δ of each variable (bucket) **(B)**. The PLS–DA model **(C)** and variable importance in projection (VIP) variables **(D)** for the four groups (*r*^2^ = 0.70, *q*^2^ = 0.43).

Blood serum samples were analyzed using two types of ^1^H NMR experiments, the basic ^1^H spectrum (single pulse-acquire), and the CPMG pulse sequence. The basic ^1^H spectrum exhibits all of the signals from both small metabolites and macromolecules resulting in an uneven baseline and the overlap of various signals from different compounds. The CPMG pulse sequence, on the other hand, suppresses the broad signals from macromolecules, namely lipids and proteins, resulting in clear peaks and a very well-defined baseline, thereby allowing better characterization and assignment of the signals arising from the small metabolites (Figure 5). A number of metabolites were able to be identified and quantified: (1) isoleucine, (2) leucine, (3) valine, (4) β-hydroxybutyrate, (5) lactate, (6) threonine, (7) alanine, (8) acetate, (9) proline, (10) glutamate, (11) glutamine, (12) methionine, (13) malate, (14) creatine, (15) choline, (16) phosphorylcholine/glycerophosphocholine, (17) taurine, (18) glycine, (19) serine, (20) water, (21) α-glucose, and (22) fumarate. Due to the breadth of the lipid signals, lipid signals were assessed and designated by moieties only: (L1) lipid methyls, (L2) lipid aliphatic chain, (L3) lipid β-methylenes, (L4) lipid allylic methylenes, (L5) lipid α-methylenes, (L6) lipid polyunsaturated allylic methylenes, and (L7) lipid alkenes (31). Figure 6A depicts the scores plot for the comparison of the CPMG spectra between control groups. Each sample is plotted according to the scores for PC1 and PC2, values that are calculated through the loadings plot (Figure 6B) where each dot represents a data point (a spectral bucket). Although the CPMG and the basic ^1^H (Figures 6C,D) provide slightly different information, the score plots are similar and WT and Ncf1 groups still overlap. However, considering the WT and Ncf1 separately, on the basis of the PCA for the Ncf1 mice, the control group is separable from the DSS-induced groups with the recovery group brought closer to the control group (Figures 7B,D). This contrasts with WT mice which present overlapping metabolomics profiles between control, recovery, and second colitis-induced groups (Figures 7A,C). Given these differences, a plot of all DSS-induced groups was prepared to see if the metabolic profile is different between WT and Ncf1 mice. The unsupervised PCA (Figures 8A,B) still shows some group overlap, but for this data set we were able to calculate a valid PLS–DA model (*r*^2^ = 0.70, *q*^2^ = 0.43) (Figures 8C,D). While the recovery groups drifted toward a common metabolic profile, the Ncf1 and WT groups after the second DSS-induction colitis are distinct from each other with lower blood glucose [variable importance in projection (VIPs) at 3.23, 3.37, 3.41, 3.52, and 5.22] and higher lactate levels (VIPs at 1.31, 4.10, and 4.15) on the Ncf1 group.

### Correlation Between Blood Serum Metabolites and Clinical Parameters

The metabolites assigned by NMR were quantified from both ^1^H and CPMG spectra and were correlated with clinical parameters. Due to the broad signals of the lipids in the ^1^H spectra, lipid signals were assessed and designated by moieties relative to the lipid methyl group (L1). Regarding the dysplasia score and the metabolome, we could only find weak-to-moderate correlations in pooled samples (Table 2). Analyzing in more detail, these correlations are only statistically significant for isoleucine, lactate, and proline in the Ncf1 group.

**Table 2:** Summary of all statistically significant Spearman correlations for dysplasia score vs metabolite concentrations considering either all mice pooled together or Ncf1-mutant (Ncf1) vs wild-type (WT) groups.

Spearman correlations		Dysplasia score		
		All groups (*n* = 44)	Ncf1 (*n* = 22)	WT (*n* = 22)
Glucose	Correlation coefficient	−0.342*	−0.344	−0.199
	Sig. (two-tailed)	0.023	0.117	0.376
L3/L1	Correlation coefficient	−0.353*	−0.321	−0.379
	Sig. (two-tailed)	0.019	0.145	0.082
L4/L1	Correlation coefficient	0.408**	0.228	0.131
	Sig. (two-tailed)	0.006	0.308	0.561
Leucine	Correlation coefficient	0.471**	0.446*	0.417
	Sig. (two-tailed)	0.001	0.037	0.053
Isoleucine	Correlation coefficient	0.511**	0.513*	0.175
	Sig. (two-tailed)	0.000	0.015	0.437
Lactate	Correlation coefficient	0.409**	0.517*	0.136
	Sig. (two-tailed)	0.006	0.014	0.545
Proline	Correlation coefficient	0.457**	0.520*	0.213
	Sig. (two-tailed)	0.002	0.013	0.341

Significant correlations are indicated by asterisks: *p < 0.05; **p < 0.01.

As regards the inflammation score, the pooled data set only presents weak-to-moderate correlations (Table 3). However, the inflammation score seems to have a more profound effect on the WT mice blood serum metabolome, with seven moderate-to-strong statistically significant correlations: positive for L2/L1, L6/L1, and L7/L1 and negative for L3/L1, valine, acetate, and choline while the Ncf1 mice blood serum metabolome only depicts a moderate negative correlation with glucose.

**Table 3:** Summary of all statistically significant Spearman correlations for inflammation score vs metabolites concentrations considering either all mice pooled together or Ncf1-mutant (Ncf1) vs wild-type (WT) groups.

Spearman correlations		Inflammation score		
		All groups (*n* = 44)	Ncf1 (*n* = 22)	WT (*n* = 22)
Glucose	Correlation coefficient	−0.394**	−0.550**	−0.168
	Sig. (two-tailed)	0.008	0.008	0.456
L2/L1	Correlation coefficient	0.457**	0.164	0.659**
	Sig. (two-tailed)	0.002	0.465	0.001
L3/L1	Correlation coefficient	−0.470**	−0.242	−0.777**
	Sig. (two-tailed)	0.001	0.278	0.000
L4/L1	Correlation coefficient	0.364*	0.313	−0.032
	Sig. (two-tailed)	0.015	0.157	0.887
L6/L1	Correlation coefficient	0.470**	0.384	0.527*
	Sig. (wo-tailed)	0.001	0.078	0.012
L7/L1	Correlation coefficient	0.530**	0.359	0.645**
	Sig. (two-tailed)	0.000	0.101	0.001
Leucine	Correlation coefficient	0.346*	0.323	0.157
	Sig. (two-tailed)	0.021	0.142	0.487
Isoleucine	Correlation coefficient	0.428**	0.419	0.016
	Sig. (two-tailed)	0.004	0.052	0.943
Valine	Correlation coefficient	−0.241	−0.088	−0.578**
	Sig. (two-tailed)	0.116	0.696	0.005
Acetate	Correlation coefficient	−0.208	0.012	−0.561**
	Sig. (two-tailed)	0.175	0.957	0.007
Glutamine	Correlation coefficient	−0.298*	−0.098	−0.302
	Sig. (two-tailed)	0.049	0.665	0.173
Choline	Correlation coefficient	−0.197	−0.065	−0.477*
	Sig. (two-tailed)	0.200	0.773	0.025
PhosphoCholine	Correlation coefficient	−0.322*	−0.336	−0.109
	Sig. (two-tailed)	0.033	0.126	0.630
Proline	Correlation coefficient	0.321*	0.388	−0.056
	Sig. (two-tailed)	0.034	0.074	0.804

Significant correlations are indicated by asterisks: *p < 0.05; **p < 0.01.

Weight variation [defined as the percentage of weight loss (negative) or gain (positive) over the course of the experiment relative to the baseline] was only weakly to moderately correlated in the pooled group but when separated into WT and Ncf1 groups, significant correlations arise, mostly for the Ncf1 group (Table 4). With the exception of glucose which is positively correlated with weight gain, all other significant correlations are negative, namely L4/L1, L6/L1, L7/L1, leucine, lactate, creatine, and proline.

**Table 4:** Summary of all statistically significant Spearman correlations for weight variation vs metabolites concentrations considering either all mice pooled together or Ncf1-mutant (Ncf1) vs wild-type (WT) groups.

Spearman correlations		Weight variation		
		All groups (*n* = 44)	Ncf1 (*n* = 22)	WT (*n* = 22)
Glucose	Correlation coefficient	0.359*	0.424*	0.149
	Sig. (two-tailed)	0.017	0.049	0.509
L2/L1	Correlation coefficient	−0.301*	−0.248	−0.383
	Sig. (two-tailed)	0.047	0.267	0.079
L3/L1	Correlation coefficient	0.340*	0.415	0.207
	Sig. (two-tailed)	0.024	0.055	0.355
L4/L1	Correlation coefficient	−0.527**	−0.427*	0.010
	Sig. (two-tailed)	0.000	0.048	0.966
L6/L1	Correlation coefficient	−0.518**	−0.519*	−0.444*
	Sig. (two-tailed)	0.000	0.013	0.038
L7/L1	Correlation coefficient	−0.418**	−0.514*	−0.449*
	Sig. (two-tailed)	0.005	0.014	0.036
Leucine	Correlation coefficient	−0.353*	−0.490*	−0.156
	Sig. (two-tailed)	0.019	0.021	0.488
Isoleucine	Correlation coefficient	−0.565**	−0.632**	−0.193
	Sig. (two-tailed)	0.000	0.002	0.389
Lactate	Correlation coefficient	−0.477**	−0.443*	−0.384
	Sig. (two-tailed)	0.001	0.039	0.078
Creatine	Correlation coefficient	−0.266	−0.433*	−0.208
	Sig. (two-tailed)	0.081	0.044	0.353
Proline	Correlation coefficient	−0.511**	−0.600**	−0.293
	Sig. (two-tailed)	0.000	0.003	0.186

Significant correlations are indicated by asterisks: *p < 0.05; **p < 0.01.

Colon length was diminished in the treated groups (Figure 1A) and this marker shows stronger correlations with blood serum metabolites for the WT group (positive for L3/L1, valine, and acetate and negative for L2/L1, L6/L1, L7/L1, and GPC/Cho) than for the Ncf1 group (negative for leucine and isoleucine) (Table 5).

**Table 5:** Summary of all statistically significant Spearman correlations for colon length vs metabolites concentrations considering either all mice pooled together or Ncf1 (Ncf1) vs wild-type (WT) groups.

Spearman correlations		Colon length		
		All groups (*n* = 43)	Ncf1 (*n* = 22)	WT (*n* = 21)
L2/L1	Correlation coefficient	−0.513**	−0.212	−0.748**
	Sig. (two-tailed)	0.000	0.343	0.000
L3/L1	Correlation coefficient	0.574**	0.418	0.735**
	Sig. (two-tailed)	0.000	0.053	0.000
L6/L1	Correlation coefficient	−0.666**	−0.510*	−0.738**
	Sig. (two-tailed)	0.000	0.015	0.000
L7/L1	Correlation coefficient	−0.669**	−0.471*	−0.830**
	Sig. (two-tailed)	0.000	0.027	0.000
GPC/Cho	Correlation coefficient	−0.258	−0.133	−0.531*
	Sig. (two-tailed)	0.095	0.556	0.013
Leucine	Correlation coefficient	−0.420**	−0.443*	−0.283
	Sig. (two-tailed)	0.005	0.039	0.213
Isoleucine	Correlation coefficient	−0.338*	−0.512*	0.011
	Sig. (two-tailed)	0.026	0.015	0.962
Valine	Correlation coefficient	0.245	−0.053	0.541*
	Sig. (two-tailed)	0.113	0.814	0.011
Acetate	Correlation coefficient	0.295	0.045	0.555**
	Sig. (two-tailed)	0.055	0.842	0.009

Significant correlations are indicated by asterisks: *p < 0.05; **p < 0.01.

For spleen weight, several weak-to-moderate correlations are present in the pooled data set (Table 6). When analyzing groups by genotype, the WT group shows strong positive correlations for the lipid moieties L2/L1, L3/L1, L6/L1, and L7/L1 and negative moderate correlations for valine, acetate, and choline. Conversely, spleen weight for the Ncf1 group only achieves moderate correlations, positive for L2/L1 and L7/L1 and negative for glucose, glutamine, phosphocholine, and glycine.

**Table 6:** Summary of all statistically significant Spearman correlations for spleen weight vs metabolites concentrations considering either all mice pooled together or Ncf1-mutant (Ncf1) vs wild-type (WT) groups.

Spearman correlations		Spleen weight		
		All groups (*n* = 42)	Ncf1 (*n* = 22)	WT (*n* = 20)
Glucose	Correlation coefficient	−0.291	−0.463*	−0.059
	Sig. (two-tailed)	0.062	0.030	0.806
L2/L1	Correlation coefficient	0.550**	0.479*	0.660**
	Sig. (two-tailed)	0.000	0.024	0.002
L3/L1	Correlation coefficient	−0.468**	−0.292	−0.654**
	Sig. (two-tailed)	0.002	0.188	0.002
L6/L1	Correlation coefficient	0.475**	0.330	0.578**
	Sig. (two-tailed)	0.001	0.134	0.008
L7/L1	Correlation coefficient	0.591**	0.523*	0.693**
	Sig. (two-tailed)	0.000	0.013	0.001
Valine	Correlation coefficient	−0.283	−0.243	−0.472*
	Sig. (two-tailed)	0.070	0.277	0.036
Acetate	Correlation coefficient	−0.370*	−0.361	−0.504*
	Sig. (two-tailed)	0.016	0.099	0.024
Glutamine	Correlation coefficient	−0.428**	−0.489*	−0.323
	Sig. (two-tailed)	0.005	0.021	0.164
Choline	Correlation coefficient	−0.395**	−0.510*	−0.456*
	Sig. (two-tailed)	0.010	0.015	0.043
PhosphoCholine	Correlation coefficient	−0.402**	−0.491*	−0.247
	Sig. (two-tailed)	0.008	0.020	0.295
Glycine	Correlation coefficient	−0.332*	−0.489*	−0.119
	Sig. (two-tailed)	0.032	0.021	0.618

Significant correlations are indicated by asterisks: *p < 0.05; **p < 0.01.

## Discussion

Models based on DSS-induced colitis are commonly used to experimentally address inflammation-associated carcinogenesis in different mouse strains (27, 32). Several studies report either a carcinogenic path dependent on longer periods or multiple cycles of DSS induction or use carcinogenic compounds to reduce the exposure periods (33–36). This contrasts with our model which develops adenocarcinoma with only two induction cycles, thereby allowing us to understand the peculiarities of epithelial morphology alterations and how they depend on ROS production. Furthermore, our model challenges the paradigm that ROS are promoters of inflammation-dependent carcinogenesis, thus allowing the possibility of studying how ROS deficiency impacts on systemic metabolomic and lipid remodeling. We show that Ncf1 mice lacking ROS production developed colonic distal high-grade dysplasia after a single 7-day exposure to 3% DSS in drinking water followed by a 14-day resting period, in contrast to the low-grade dysplasia in the colon of ROS-competent WT mice. Furthermore, after a 21-day resting period we observed lower inflammatory reparation and high-grade dysplasia and invasive well-differentiated adenocarcinoma in the Ncf1 mice while in the WT mice, dysplasia was also prominent without malignant invasion. The presence of adenocarcinoma has severely compromised.

In human colon cancer, the inactivation of the adenomatous polyposis coli (APC) gene is present in the large majority of patients, with concomitant stabilization and accumulation of β-catenin especially in the epithelium [reviewed in Ref. (37)]. Recently, it has been demonstrated that in colon carcinoma patients the T cells present in the inflammatory foci in the colon express elevated levels of β-catenin, and mice with over-activation of β-catenin develop chronic colonic inflammation and subsequent carcinogenesis (38). In a previous study, we could not detect any alteration on the expression levels of the *Apc* gene in Ncf1 mice bread either in SPF or germ-free conditions (39). However, we observe an accumulation of β-catenin expressing lymphocytes in the inflammatory foci of the Ncf1 colon near well-differentiated adenocarcinoma. In our previous study on acute colitis, we have seen a massive infiltration of T cells into those inflammatory foci (25). Hence, it is worth exploring further the possible link between ROS-dependent failure in tolerance induction in Ncf1 T cells and dysregulation of β-catenin expression, which may contribute to promote chronic inflammation and ultimately carcinogenesis.

Even though both unchallenged WT and Ncf1 mice exhibit a similar blood serum metabolic fingerprint, their response to DSS exposure led to distinct metabolomic rearrangements. The metabolomics approach highlighted lower blood glucose and higher lactate levels in the Ncf1 mice, which may reflect compromised intestinal nutrient absorption (40). This is supported by the observation of bloody feces, reduced colon length, and reduced body weight, all distinct signs of colitis onset. Regarding clinical parameters and blood serum metabolites in Ncf1 mice, we consistently found lower glucose and/or higher lactate levels correlating with dysplasia and inflammation score and weight variation.

Another interesting correlation concerns the blood plasma lipid remodeling upon the development of colitis. Although WT DSS-induced colitis mice exhibited less severe histopathological changes, the blood serum metabolome displayed an increase in the fatty acids as evidenced by gains in L2, L6, and L7 and exhibited moderate-to-strong correlations with inflammation score, weight variation, colon length, and spleen weight. The Ncf1 mice exhibited a similar response, though with lower correlation coefficients. Considering that DSS-induced colitis may alter hepatic metabolism (41), these lipid rearrangements can modulate the immune response, and the inflammatory process itself (42, 43). Moreover, a broader systemic metabolic remodeling might be in play, with recent studies relating dietary patterns and hormonal regulation (43–45). Strong localized inflammation may release cytokines, such as IL-6 and C-reactive protein, into the circulatory system and activate a systemic response by the sympathetic nervous system and hypothalamic–pituitary–adrenal axis (44, 46). This response may generate a hormonally induced metabolic shift, including possible peripheral insulin resistance and inhibiting the storage of energy-rich substrates in the liver, muscle, and adipocytes. The increased levels of circulatory energy-rich substrates may fuel activated immune cells, which can be crucial in the context of IBD, where nutrient absorption is directly impaired. Furthermore, the increase of circulatory unsaturated fatty acids may also exert a pro-inflammatory positive feedback loop (43, 44). However, this is still a fairly controversial notion given the plethora of lipids classes where microbiota-derived short-chain fatty acids and unsaturated lipids of dietary origin seem to promote anti-inflammatory effects (47, 48) while an altered polyunsaturated lipid profile in colonic mucosa correlates with the severity of inflammation (49).

Besides lipid rearrangements, DSS-induced Ncf1 mice also displayed an increase in a few blood serum amino acids, e.g., leucine, isoleucine, and proline. This was probably related to protein breakdown for energetic purposes, a conjecture supported by the observed low-blood glucose levels and weight loss (40). These highlighted metabolic responses are consonant with those previously described in DSS-induced colitis protocols (50, 51).

Despite a few studies on animal models and CGD patients showing that ROS have a crucial role in immune regulation (19, 52, 53), oxidative burst and the concomitant production of ROS are still regarded mainly as pro-inflammatory events. However, in this study we stress their protective role against chronic inflammation and tumor development. The lack of capacity of NOX2 to generate oxidative burst in the Ncf1 model enhanced the DSS-induced colitis symptoms as far as cancer onset.

Another feature of NOX2 activity is the consumption of reducing equivalents, either as cofactor for the reaction itself or by ROS-scavenging enzymes. Taken together with the transient O_2_ depletion and HIF signaling, we might expect metabolic rearrangements in several pathways, namely for mitochondrial and lipogenic activities. Unfortunately, only a very few studies focused on the metabolic changes in colitis mucosa. The pentose phosphate pathway (PPP) is a major NADPH generator, especially in O_2_ deficient environments. *TP53*-inducible glycolysis and apoptosis regulator (TIGAR) may redirect glycolytic metabolism toward PPP, but also present divergent regenerative or tumorigenic outcomes, while in DSS-induced colitis, TIGAR dampens mild oxidative stress, promoting cellular regeneration; however, in cancer with deregulated p53 responses, it enhances proliferation by limiting excessive ROS generation and providing nucleotides (54). Thus, it will be interesting to understand whether this mechanism is impaired in Ncf1 mice, and if intact NOX2 activity may promote TIGAR activity to maintain NADPH levels through PPP thereby adding another protective layer.

Furthermore, lipogenesis may be impaired given the requirement of NADPH by fatty acid synthase, resulting in disturbed mucosal lipid metabolism. Again, the scarce literature only mentions a fatty acid synthase increased expression in both DSS-induced colitis mucosa and colorectal cancer (55, 56) and our work only focused on the systemic blood serum metabolome.

In our study, the lack of inflammatory-derived ROS on Ncf1 DSS-induced colitis model was sufficient to develop adenocarcinoma and exhibit a different blood plasma lipid profile. Even though oxidative burst is responsible for several pro-inflammatory signaling events, it is also required for initiating resolution of inflammatory processes in an ROS-mediated self-control mechanism. Although mainstream lines of research focus on ROS generation, the answer for this paradoxical mechanism may lie in the substrates, the transiently variable O_2_ concentrations in the microenvironment, and the cofactor NADPH. Further research on the metabolic pathways involved, especially for the regeneration of NADPH through PPP and *de novo* lipogenesis, may provide new targets which until now have been disregarded.

The present study also reveals some, as yet unaddressed, peculiarities of the development of colon adenocarcinoma. In the histological analysis of the colon from our model, we followed the 2010 WHO classification of tumors of the digestive system (26) applying the human nomenclature for low-grade dysplasia and high-grade dysplasia/intraepithelial neoplasia. The present study shows that villous/superficial papillary adaptation of the flat mucosa was not relevant to adenocarcinoma morphology and that the inflammation process in Ncf1 colon form a spectrum from acute to the adaptive presence of inflammatory cells. We show that basal glands, the hallmark of the WT response, were mostly insignificant in Ncf1 mice, which displayed installed dysplasia, quickly evolving to invasive well-differentiated adenocarcinoma. The WHO nomenclature and other studies recognize several patterns for colonic tumors: adenocarcinoma, mucinous adenocarcinoma, signet-ring cell carcinoma, and undifferentiated carcinoma (26, 57). Our model was only capable of inducing well-differentiated tubular/glandular adenocarcinoma, with sporadic mucinous cells hyperplasia in the foci of high-grade dysplasia. These important findings suggest that hyperplastic villous patterns, either in the mucosa or in villous and tubulo-villous adenomas, may correspond to an epithelial adaptive modification also observed in gastric peptic ulcer re-epithelization (64). ROS influence in the studied carcinogenesis was underlined by the morphological alterations that were more prominent in Ncf1 mice, whereas the presence of ROS may allow mucosal adaptation in WT mice.

This work brings new data on the relevance of an intact ROS production for an effective resolution of chronic colon inflammation thus helping prevent degeneration into a carcinogenic process with systemic metabolomic and lipidomic shifts.

## Ethics Statement

This study was carried out in accordance with the recommendations of EU legislation for experimental animal welfare. The protocol was approved by the internal FFUC Animal Facility Ethics Committee.

## Author Contributions

LT, AX, JG, and KK performed experimental work and participated in the study design. AX, JG, and LT performed data analysis. LT, KK, RH, LC, RC, and MS-C contributed with experimental design, data acquisition/analysis, and writing.

## Conflict of Interest Statement

The authors declare that the research was conducted in the absence of any commercial or financial relationships that could be construed as a potential conflict of interest. The reviewer EV declared a shared affiliation, though no other collaboration, with one of the authors RH to the handling Editor.

## Abbreviations

DSS, dextran sulfate sodium; HE, hematoxylin/eosin; HIF, hypoxia-inducible factor; IBD, inflammatory bowel disease; Ncf1, Ncf1-mutant; NADPH, nicotinamide adenine dinucleotide phosphate; NOX, nicotinamide adenine dinucleotide phosphate oxidase; PCA, principal component analysis; PLS–DA, partial least squares–discriminant analysis; PPP, pentose phosphate pathway; NMR, nuclear magnetic resonance; ROS, reactive oxygen species; TIGAR, *TP53*-inducible glycolysis and apoptosis regulator; UC, ulcerative colitis; VIP, variable importance in projection; WT, wild-type; WHO, World Health Organization.
